# NMR metabolomic modeling of age and lifespan: A multicohort analysis

**DOI:** 10.1111/acel.14164

**Published:** 2024-04-18

**Authors:** Chung-Ho E. Lau, Maria Manou, Georgios Markozannes, Mika Ala-Korpela, Yoav Ben-Shlomo, Nish Chaturvedi, Jorgen Engmann, Aleksandra Gentry-Maharaj, Karl-Heinz Herzig, Aroon Hingorani, Marjo-Riitta Järvelin, Mika Kähönen, Mika Kivimäki, Terho Lehtimäki, Saara Marttila, Usha Menon, Patricia B. Munroe, Saranya Palaniswamy, Rui Providencia, Olli Raitakari, Amand Floriaan Schmidt, Sylvain Sebert, Andrew Wong, Paolo Vineis, Ioanna Tzoulaki, Oliver Robinson

**Affiliations:** 1 MRC Centre for Environment and Health, Department of Epidemiology and Biostatistics, School of Public Health, Imperial College London, London, UK; 2 Department of Hygiene and Epidemiology, University of Ioannina Medical School, Ioannina, Greece; 3 Systems Epidemiology, Faculty of Medicine, University of Oulu, Oulu, Finland; 4 Research Unit of Population Health, Faculty of Medicine, University of Oulu, Oulu, Finland; 5 Biocenter Oulu, University of Oulu, Oulu, Finland; 6 NMR Metabolomics Laboratory, School of Pharmacy, Faculty of Health Sciences, University of Eastern Finland, Kuopio, Finland; 7 Population Health Sciences, University of Bristol, Bristol, UK; 8 MRC Unit for Lifelong Health and Ageing at UCL, University College London, London, UK; 9 UCL Institute of Cardiovascular Science, Population Science and Experimental Medicine, Centre for Translational Genomics, London, UK; 10 MRC Clinical Trials Unit at UCL, Institute of Clinical Trials and Methodology, University College London, London, UK; 11 Department of Women’s Cancer, Elizabeth Garrett Anderson Institute for Women’s Health, University College London, London, UK; 12 Institute of Biomedicine and Internal Medicine, Biocenter of Oulu, Medical Research Center Oulu, Oulu University Hospital, Faculty of Medicine, Oulu University, Oulu, Finland; 13 Department of Pediatric Gastroenterology and Metabolic Diseases, Poznan University of Medical Sciences, Poznan, Poland; 14 Department of Life Sciences, College of Health and Life Sciences, Brunel University London, London, UK; 15 Department of Clinical Physiology, Tampere University Hospital, Tampere, Finland; 16 Faculty of Medicine and Health Technology, Tampere University, Tampere, Finland; 17 Brain Sciences, University College London, London, UK; 18 Faculty of Medicine and Health Technology and Finnish Cardiovascular Research Center Tampere, Tampere University, Tampere, Finland; 19 Department of Clinical Chemistry Fimlab Laboratories, Tampere, Finland; 20 Molecular Epidemiology, Faculty of Medicine and Health Technology, Tampere University, Tampere, Finland; 21 Gerontology Research Center (GEREC), Tampere University, Tampere, Finland; 22 William Harvey Research Institute, Barts and the London Faculty of Medicine and Dentistry, Queen Mary University of London, London, UK; 23 National Institute of Health and Care Research, Barts Cardiovascular Biomedical Research Centre, Queen Mary University of London, London, UK; 24 Institute of Health Informatics Research, University College London, London, UK; 25 Barts Heart Centre, Barts Health NHS Trust, London, UK; 26 Centre for Population Health Research, University of Turku and Turku University Hospital, Turku, Finland; 27 Research Centre of Applied and Preventive Cardiovascular Medicine, University of Turku, Turku, Finland; 28 Department of Clinical Physiology and Nuclear Medicine, Turku University Hospital, Turku, Finland; 29 Institute of Cardiovascular Science, Faculty of Population Health Sciences, University College London, London, UK; 30 Department of Cardiology, Amsterdam Cardiovascular Science, Amsterdam University Medical Centers, University of Amsterdam, Amsterdam, The Netherlands; 31 UCL BHF Research Accelerator Centre, London, UK; 32 Biomedical Research Foundation, Academy of Athens, Athens, Greece; 33 Ageing Epidemiology (AGE) Research Unit, School of Public Health, Imperial College London, London, UK

**Keywords:** aging, biological age, cohort study, metabolome, metabolomics, molecular epidemiology, mortality, NMR, population aging

## Abstract

Metabolomic age models have been proposed for the study of biological aging, however, they have not been widely validated. We aimed to assess the performance of newly developed and existing nuclear magnetic resonance spectroscopy (NMR) metabolomic age models for prediction of chronological age (CA), mortality, and age-related disease. Ninety-eight metabolic variables were measured in blood from nine UK and Finnish cohort studies (*N* ≈31,000 individuals, age range 24–86 years). We used nonlinear and penalized regression to model CA and time to all-cause mortality. We examined associations of four new and two previously published metabolomic age models, with aging risk factors and phenotypes. Within the UK Biobank (*N* ≈102,000), we tested prediction of CA, incident disease (cardiovascular disease (CVD), type-2 diabetes mellitus, cancer, dementia, and chronic obstructive pulmonary disease), and all-cause mortality. Seven-fold cross-validated Pearson’s *r* between metabolomic age models and CA ranged between 0.47 and 0.65 in the training cohort set (mean absolute error: 8–9 years). Metabolomic age models, adjusted for CA, were associated with C-reactive protein, and inversely associated with glomerular filtration rate. Positively associated risk factors included obesity, diabetes, smoking, and physical inactivity. In UK Biobank, correlations of metabolomic age with CA were modest (*r* = 0.29–0.33), yet all metabolomic model scores predicted mortality (hazard ratios of 1.01 to 1.06/metabolomic age year) and CVD, after adjustment for CA. While metabolomic age models were only moderately associated with CA in an independent population, they provided additional prediction of morbidity and mortality over CA itself, suggesting their wider applicability.

## Abbreviations

ALSPAC: Avon Longitudinal Study of Parents and Children
ApoB: apolipoprotein B
BMI: body mass index
BWHHS: British Women’s Health Heart Study
CA: chronological age
CAPS: Caerphilly Prospective Study
CI: confidence interval
COPD: chronic obstructive pulmonary disease
CRP: C-reactive protein
CVD: cardiovascular disease
DBP: diastolic blood pressure
DHA: docosahexaenoic acid
eGFR: estimated glomerular filtration rate
FDR: false discovery rate
FEV1: forced expiratory volume in first second.
HDL: high-density lipoprotein
IDL: intermediate-density lipoprotein
LDL: low-density lipoprotein
MAE: mean absolute error
MARS: multivariate adaptive regression splines.
NFBC1966: The 1966 Northern Finland Birth Cohort
NMR: nuclear magnetic resonance
NSHD: MRC National Survey of Health and Development
SABRE: Southhall and Brent Revisited
SBP: systolic blood pressure
TG: triglycerides
UCLEB: UCL-LSHTM-Edinburgh-Bristol Consortium
UKB: UK Biobank
UKCTOCS: The Collaborative UK Ovarian Cancer Screening Trial
VIF: variable inflation factor
VIP: variable importance of projection
VLDL: very low-density lipoprotein
WHII: Whitehall II
YFS: Young Finns Study.

## 1 Introduction

Aging can be broadly defined as a time-dependent decline of functional capacity and stress resistance, associated with increased risk of morbidity and mortality (Burkle et al., 2015). The rate of aging may vary between individuals and groups due to both environmental stressors, including lifestyle, social adversity (Stringhini et al., 2018), and genetic factors (McDaid et al., 2017). This divergence in the rate of aging can lead to discrepancies between “biological” and chronological age. Markers of biological age may allow improved prediction of health- and life-span than chronological age itself and allow identification of vulnerable individuals (Ferrucci et al., 2018).

Recently, high throughput “omic” methods, which provide simultaneous quantification of sets of multiple molecular features, have been used to develop “biological clocks” that provide a global measure of changes with age at the molecular level (Rutledge et al., 2022). Metabolomics, the global profiling of small molecules with a molecular weight of <1500 Da in the body, has emerged as a promising analytical approach for assessing molecular changes with age at the population level (Panyard et al., 2022; Robinson & Lau, 2023). Overall, the rate of metabolism declines with age (Pontzer et al., 2021) and more specifically all aging hallmarks are expected to have detectable effects on the metabolome, including hallmarks of cellular aging such as nutrient sensing, mitochondrial dysfunction, and altered intracellular communication which directly relate to metabolic alterations (Lopez-Otin et al., 2016; Nilsson et al., 2019).

We previously reported a metabolomic clock based on untargeted mass-spectrometry (Robinson et al., 2020) in a cohort of around 2000 people, observing strong prediction of chronological age (CA) in internal test sets, associations between metabolomic age and noncommunicable disease risk factors, and enrichment of known aging pathways among model predictors. However, this clock cannot be easily applied to other datasets due the untargeted nature of the data used. An alternative approach is to use nuclear magnetic resonance spectroscopy (NMR), a metabolomic platform that provides more precise quantification enabling more straight-forward application across studies. van den Akker et al. (2020) used the Nightingale platform of NMR-based metabolomics in blood, to linearly model CA in a large Dutch Biobank sample of 25,000 people from 26 cohorts (age range 18–85), finding their metabolomic age measure was predictive of cardiovascular events and mortality. While their metabolomic age measure was strongly correlated with CA in an internal test set, internal validation may provide over-optimistic assessments of model performance (Rodriguez-Perez et al., 2018) and their measure remains to be widely tested in external datasets.

When developing biological age clocks, two divergent approaches have emerged: training on CA, which will identify molecular features and pathways that change with CA but may be less sensitive for assessing age-related health status; and training on lifespan (i.e., time-to-all-cause mortality) which may more accurately reflect one's age-related health status, yet will also assess early-effects of disease in addition to intrinsic biological aging mechanisms (Bernabeu et al., 2023). In this regard, the multivariable NMR-based metabolite score of all-cause mortality developed by Deelen et al. in 44,000 people may be considered a biological age marker as it explicitly assesses remaining lifespan. The model was found to have greater predictive accuracy than a model containing conventional risk factors (Deelen et al., 2019).

In the present study, we aimed to develop new NMR-based metabolomic aging models, incorporating variable selection, non-linear modeling, and lifespan information, within nine UK and Finnish cohorts of 38,000 samples covering most of adult life. To judge their potential utility for the assessment of differential metabolic aging, we assessed and compared their associations with aging risk factors, phenotypes, and cardiovascular disease and mortality incidence. Finally, to understand the reproducibility of the NMR-based metabolomic aging models, we tested their performance, alongside the previously published Akker et al. and Deelen et al. models, in the UK Biobank (UKB, *N*= 102,000 individuals) for the prediction of CA, mortality, and a diverse range of age-related diseases.

## 2 Methods

### 2.1 Study population

The study included six British cohorts participating in the UCL-LSHTM-Edinburgh-Bristol (UCLEB) Consortium (Shah et al., 2013): The MRC National Survey of Health and Development (NSHD), the Caerphilly Prospective Study (CAPS), the British Women’s Heart and Health Study (BWHHS) (Lawlor et al., 2003), the Southhall and Brent Revisited Study (SABRE), the Whitehall-II study (WHII) (Marmot & Brunner, 2005), and the UK Collaborative Trial of Ovarian Cancer Screening Longitudinal Women’s Cohort (UKCTOCS) (Jacobs et al., 2016). Two studies from Finland were included: The 1966 Northern Finland Birth Cohort (NFBC1966) and the Young Finns Study (YFS). In addition, we included the British Avon Longitudinal Study of Parents and Children (Boyd et al., 2013), which included samples from fathers (ALSPAC-partners) (Northstone et al., 2023) and mothers (ALSPAC-mothers) (Fraser et al., 2013). ALSPAC-partners and ALSPAC-mothers were considered as different cohorts and analysed separately. Longitudinal samples were available from SABRE at two timepoints (SABRE1 and SABRE2) which were collected between 1988–1991 and 2008–2011. Follow-up samples were available from NFBC1966 at two timepoints when participants were 31 (NFBC1966 [31 years]) and 46 years old (NFBC1966 [46 years]), and longitudinal YFS samples available were followed-up in 2001 (YFS2001), 2007 (YFS2007) and 2011 (YFS2011). These follow-up samples were analysed separately since follow-up clinics and sampling were conducted on separate occasions. UKB study samples (*N* ≈102,000) were used for model validation in this study. Ethical approval for each cohort study was obtained from the Local Research Ethics Committees. Informed consent for the use of data collected via questionnaires and clinics and analysis of biological samples was obtained from all participants. Additionally, the current study was approved by the Imperial College Research Ethics Committee (Reference: 19IC5567). Details on individual cohort characteristics are listed in Table S1.

### 2.2 Metabolomic data acquisition and preprocessing

A high throughput ^1^H NMR spectroscopy metabolomics platform (Brainshake Ltd./Nightingale Health©, Helsinki, Finland) was applied to fasted blood serum, except in UKB where EDTA-plasma samples were used for metabolomics analysis. The assay provides concentration measurements for a range of metabolite variables including lipoprotein subclasses and individual lipids, fatty acids, glucose and various glycolysis precursors, ketone bodies, and amino acids. The NMR platform also reports on average sizes of lipoprotein particle subclasses VLDL, LDL, and HDL. Details of this platform have been published previously (Soininen et al., 2009; Wurtz et al., 2017). Two hundred and thirty-three lipid and metabolite measures were initially obtained from the assay platform, although some of the metabolic measures were frequently missing in one or more cohorts and were removed from subsequent analysis. Acetoacetate, pyruvate, glycerol, glycine, diacylglycerol, conjugated linoleic acid, and estimated description of fatty acid chain length were excluded as a result. Derived metabolic variables, including variables expressed as ratios or percentages were also excluded to limit data redundancy. Additionally, we also examined correlations of metabolic variable signals derived from Nightingale pre-2020 and post-2020 quantification protocols available for 6446 YFS samples and excluded variables with Pearson’s *r* < 0.7. The remaining 98 well quantified metabolic variable signals were considered in the main part of this study (Table S2). Multivariate outlier detection was carried out per study cohort, using the *pcout* function from R package *mvoutlier*. Since our key objective was to develop multivariable metabolomic models, a multivariate outlier detection method was chosen to identify and remove samples which behave uncharacteristically compared the rest of the observations in the multivariate space prior to analysis. The method is based on principal components analysis and observations were considered location outliers if they have been assigned a weight ≤0.1, and these were subsequently removed from the study. Six thousand, one hundred four samples were removed as a result, and the number of samples after quality control was 37,888.

Additionally, to minimize bias originated from preanalytical and analytical differences in among the study cohort datasets, we calibrated the metabolic data between cohorts and visits using methodology as described in Makinen et al. (2022) as part of the data preprocessing. Whitehall II (WHII) was a mixed-sex cohort in the middle of the age range in among our samples and was thus defined as the reference dataset in the calibration, with all other study cohort datasets were normalized against the WHII samples. During the cohort data calibration, a subset of samples of matching demographic characteristics, including age, sex, body mass index (BMI), and ethnicity were selected from both the target and the reference datasets, and scaling factors were then estimated per metabolic variable and subsequently applied to the full cohort data in the target sets. Principal component analysis was performed and the results confirm no clustering of samples by cohort could be observed in the first two principal components (Figure S1). The distribution of nontransformed values in the 98 metabolic variables were broadly normal, as determined through inspection of histograms and quantile-quantile plots (Figure S2 and S3). Subsequent sensitivity analyses, performed using log-transformed variable data, confirmed choice of data transformation had minimal effect on results.

### 2.3 Metabolome wide association study (MWAS) of age and mortality

To understand individual metabolite associations with aging, we first performed univariate analyses of metabolic variables with age and mortality. Cohort-stratified metabolome wide association study (MWAS) of age was assessed using multiple linear regression adjusted for sex, BMI, and ethnicity. Age stratified MWAS of age were performed to examine the consistency of age-metabolite associations across the life-course, and these analyses were additionally adjusted for cohort. The following age group strata were used: 20–35, 35–40, 40–45, 45–50, 50–55, 55–60, 60–65, 65–70, and >70. Multiple Cox proportional hazard regressions (*survival* R package) adjusted for CA, sex, and BMI were used to estimate the associations with mortality, within the UKCTOCS, WHII, and SABRE cohorts where this information was available. Inverse variance-weighted fixed effect meta-analyses were used to pool study cohort estimates, Benjamini & Hochberg’s false discovery rate (FDR) was used when accounting for multiple testing, with an FDR-corrected *q* < 0.05 denoting significance, and heterogeneity among the cohorts/age group strata was assessed using Cochran’s Q test and *I*^2^ using the *meta* R package. Variables with *I*^2^ values >0.75 were considered of high heterogeneity, whilst those with *I*^2^ values <0.25 were considered of low heterogeneity.

### 2.4 Multivariable predictive modeling of aging

NSHD and NFBC1966 were birth cohorts and were excluded from model training since study participants all share identical CA and would therefore likely bias the training sample set. Consequently, the training sample set consisted of 26,640 samples from eight study cohorts. To avoid problems associated with multicollinearity in model training and improve model stability, a pruned variable set was generated from the full set of predictors using sequential backward stepwise selections and the variable inflation factor (VIF), derived using *vif* function in the *car* R package, as selection criteria. Starting with all 98 predictors as model inputs, in a stepwise fashion, the variable with the largest VIF value was removed and a new model was generated with one less variable than in the previous step, until no variables had a VIF ≥5. This yielded 24 variables (Table S2), which were then used as input predictors in our multivariable CA models. Pairwise Pearson’s correlations of the 24 metabolic variables, both within the training cohort set and within UKB, are presented in Figure S4. Seven-fold cross validation and leave-one-cohort out (LOCO) validation were used to assess model stability and prediction performance during training.

#### 2.4.1 Elastic net and MARS models

Multivariable models of study CA were constructed using elastic net regression (*glmnet* and *caret* R packages), and 2nd degree multivariate adaptive regression splines (MARS, *earth*, and *caret* R packages) models. Elastic net is a versatile penalized linear regression model which simultaneously performs variable selection and modeling fitting. It is computationally efficient, suitable for highly correlated datasets, and resultant models are easily interpretable (Zou & Hastie, 2005). The alpha parameter in *glmnet* was preselected as 0.5 in the elastic net model. The MARS approach is suitable for regression problems when the relationship between predictors and response variables are nonlinear, as the model takes the form of an expansion in product spline basis functions (Friedman, 1991). A second-degree MARS model was used as it is suitable for modeling quadratic predictor-response relationships and is considered efficient when the number of model predictor variables is relatively small. Model variable importance scores (VIP) were evaluated in the training sample data using the *vip* R package.

#### 2.4.2 Study mortality score

Instead of training the metabolic data on CA, multivariable modeling was performed with survival treated as dependent variable in a penalized Cox regression. Study samples from WHII, SABRE, and UKCTOCS were used for model training, which was performed using the *glmnet* R package with alpha parameter in the elastic net model selected as 0.5. The 24 metabolite variables, and covariates comprised of age, sex, BMI, ethnicity, and cohort were included as model input predictors. After including only metabolic variable predictors and excluding contributions from other covariates, the resultant model was considered as the study mortality score, and these were subsequently scaled to the means and standard deviations of CA in the study cohort sample data to render score units in years.

#### 2.4.3 Phenotypic aging

The phenotypic aging model represented a hybrid approach, and it simultaneously incorporated metabolic information of both age and mortality into the model training process. While this model was trained on sample CA, we additionally introduced a weighted approach to allow differential predictor shrinkage based on the direction and strength of their associations with mortality in our study samples. More specifically, the differential weights on the predictors were introduced as penalty factors into the *glmnet* model. Whereas a penalty factor of 0 would suggest no shrinkage, metabolic variables with large penalty factors would be heavily penalized in the model. *P-*values obtained from proportional hazard regressions of metabolic variables on mortality were applied as model penalty factors, and in addition, variables showing opposing direction of associations with age and mortality were assigned a penalty factor of 1. This approach has the effect of enhancing the influence of metabolic variables that are closely associated with mortality/ health outcome whilst still providing a direct prediction on sample CA.

#### 2.4.4 Akker et al. and Deelen et al. models

The Akker et al. model predicts CA (in years) directly. Model weight/coefficients were extracted from their original publication (van den Akker et al., 2020). The Deelen et.al model was computed using 14 log-transformed and cohort-scaled biomarkers multiplied by their weight based on log-hazard ratios from meta-analyses as reported in Deelen et.al’s publication (Deelen et al., 2019), and subsequently summed. The resulting score was scaled to the means and standard deviations of cohort CA in the study cohort sample data to render score units in years. Acetoacetate concentrations were missing in the ALSPAC-partners and CAPS study, and these values were imputed using k-nearest neighbors method from the *impute* R package for the purpose of generating the Deelen et al. and Akker et al. model scores. The Akker et al. model was not applied to the UKB as two of the specified model variables have since been discontinued and were not available in the UKB dataset (Bizzarri et al., 2023).

Further details of the multivariable aging models are provided in the Appendix S1.

### 2.5 Covariate coding of disease risk factors and adverse health outcomes

Hypertension was defined by doctor diagnosis in the YFS and UKCTOCS cohorts, by systolic blood pressure ≥140 mm Hg or doctor diagnosis in the NFBC1966, ALSPAC-mothers, ALSPAC-partners, and SABRE cohorts, by use of antihypertensive medication or systolic blood pressure ≥140 mm Hg in the NSHD and BWHHS cohorts and by systolic blood pressure ≥140 mm Hg only in WHII. Diabetes was defined by doctor diagnosis in the YFS, ALSPAC-mothers, ALSPAC-partners, and UKCTOCS cohorts, by fasting glucose ≥7 mmol/L or doctor diagnosis in the NFBC1966, NSHD and CAPS cohorts, by fasting glucose ≥7 mmol/L, 2-h postload glucose ≥11.1 mmol/L or doctor diagnosis in the WHII cohort, by glycated hemoglobin (HbA1c) ≥ 6.6% or doctor diagnosis in the BWHHS cohort and by fasting glucose ≥7 mmol/L only in the SABRE cohort. Physical inactivity was defined as no or less than once per week of moderate/vigorous physical activity in most cohorts. For CAPS and SABRE, it was defined as the lowest tertile of calculated weekly physical activity estimates. Smoking was classified as never/former versus current smoker. Alcohol consumption was defined as no/moderate versus heavy consumption. Heavy alcohol use was defined in the NFBC1966, YFS, NSHD, WHII, CAPS, UKCTOCS, and BWHHS for men as >21 units of alcohol per week and for women as >14 units of alcohol per week. In ALSPAC-mothers and ALSPAC-partners, heavy alcohol use was defined as more than 4 times per week. Three measures of socioeconomic position (SEP) were used representing the early, mid-, and later life periods: Occupation of the participants’ fathers was classified as a manual versus nonmanual. Educational level was a binary indicator when comparing those with up to secondary-level schooling only with those with further or higher education. Current or last occupation of participants was classified as manual versus nonmanual.

Within the SABRE and UKCTOCS cohorts, coronary heart disease events were available: we have included both nonfatal and fatal myocardial infarction, revascularization, and unstable angina events in the SABRE cohort analysis, and acute coronary syndrome, myocardial infarction, angina, and other acute and chronic ischaemic heart disease events were included in the UKCTOCS analyses. Both fatal and nonfatal stroke were included in the association analyses. All-cause mortality was available in the WHII, SABRE, and UKCTOCS cohorts. Within UKB, we analysed incident all-cause mortality and cardiovascular disease (CVD), type-2 diabetes mellitus (T2DM), chronic obstructive pulmonary disease (COPD), cancer, and dementia. CVD defined as the composite of myocardial infarction (MI) cases (ST-Elevation MI and Non-ST-Elevation MI) and stroke cases (ischaemic, intracerebral hemorrhage, and subarachnoid hemorrhage).

### 2.6 Analysis of metabolomic age with aging risk factors, phenotypes, and incident health events

Diabetes, hypertension, obesity (BMI>30), physical inactivity, current smoking status, heavy alcohol consumption, education attainment, and occupation status were included in the noncommunicable disease risk factor analyses, categorized as binary variables. Six aging-related biomarkers, including systolic (SBP) and diastolic blood pressure (DBP), pulse pressure, C-reactive protein (CRP), estimated glomerular filtration rate (eGFR), and forced expiratory volume in first second (FEV1) were available from multiple, but not all cohorts. Biomarkers were univariate scaled to facilitate cross-comparison. Summary of biological aging markers data by cohort are shown in Table S1. For the analysis of associations between metabolomic aging models, aging biomarkers, and disease risk factors, linear regression models were adjusted for CA, sex, and ethnicity. To avoid including repeated samples from the same individuals from multiple follow-up visits, samples from YFS2001, YFS2007, NBFC1966 (31 years), and SABRE2 were excluded from the risk factors analysis. Cox proportional hazard regressions adjusted for CA, sex, and ethnicity were used to estimate the associations with disease and mortality incidence (*surviva*l R package), within the WHII, UKCTOCS, SABRE cohorts, and the UKB. Since all analyses were adjusted for CA, estimates can be interpreted as years of additional metabolomic age relative to CA, equivalent to formulations such as “age acceleration” and “age gap” often used within the biological clock literature. A *p* value threshold of 0.001 was chosen for the reporting of statistical significance, considering multiple testing and the number of independent tests performed. All analyses were conducted in R version 4.

## 3 Results

### 3.1 Age and lifespan associations of metabolites

**Figure 1:**
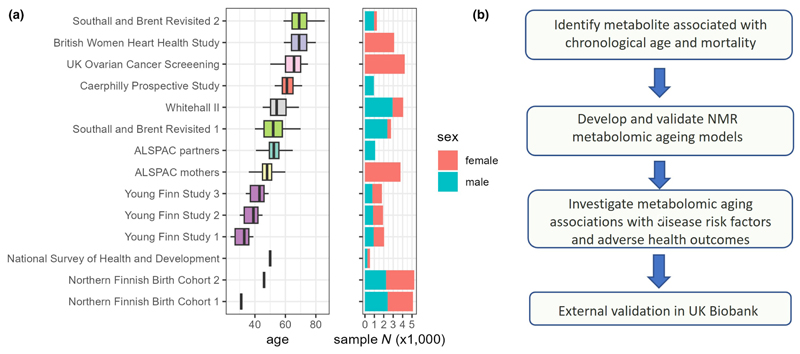
(a) Study cohorts age and sex profile (37,888 samples from 30,913 subjects, of which 26,640 samples from 22,828 subjects were used for model training). The 1966 Northern Finland Birth Cohort (NFBC1966) and National Survey of Health and Development (NSHD) are both birth cohorts, where study participants all share identical age, and were only used for risk factor association analyses. Cohort studies with multiple follow-ups were represented by the same color. (b) Current study workflow.

Analysis of metabolic aging included 26,640 samples (aged 24–86, 60% female) from 22,828 individuals in eight cohorts, including 728 and 1992 participants from the SABRE and YFS cohorts respectively, who were assessed in more than one follow-up. Individual cohort characteristics can be found in Figure 1a and Table S1.

**Figure 2:**
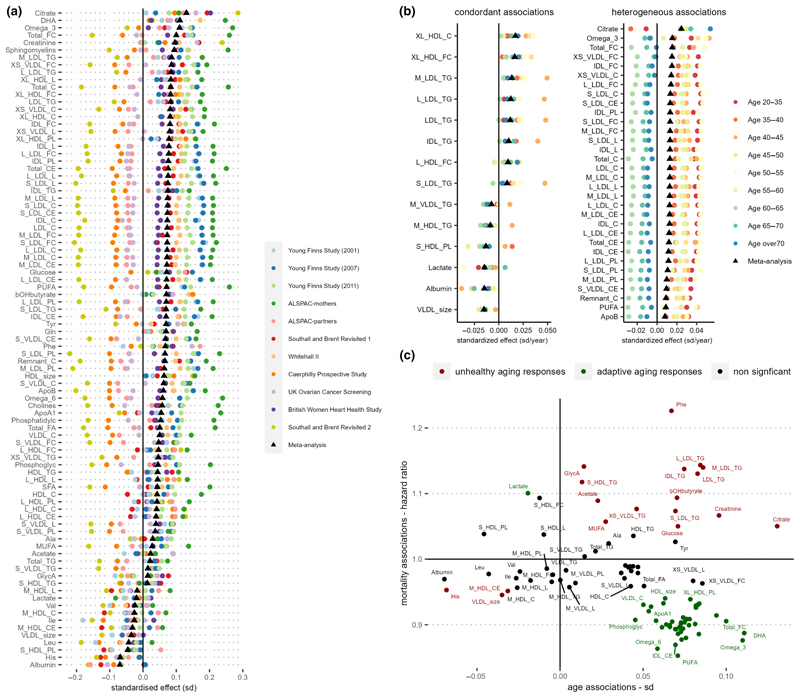
(a) Age associations with NMR metabolome by individual cohort studies. Linear regression models were adjusted for sex, BMI, and ethnicity. Metabolite variables shown were found significant after FDR correction in inverse variance-weighted fixed effect meta-analyses. (b) Meta-analysis of age-group stratified age-NMR metabolome associations. Linear regression models were first performed in the following age group strata: 20–35, 35–40, 40–45, 45–50, 50–55, 55–60, 60–65, 65–70, and >70. and models were adjusted for sex, BMI, ethnicity, and cohort. Inverse variance-weighted fixed-effect meta-analysis were then performed to pool the stratified age-group model estimates. Metabolic variables found significant with FDR *q* < 0.05 with *I*^2^ values >0.75 (high heterogeneity), or <0.25 (low heterogeneity) were shown. (c) Scatter plot of model regression coefficients of chronological age against mortality pooled hazard ratios. For mortality analysis, cohort-specific Cox proportional hazards regression models were adjusted for age, sex, BMI, and ethnicity; fixed effected meta-analysis was performed to pool together individual cohort estimates. Significant metabolic associations against both age and mortality after correcting for FDR (*q* < 0.05) were highlighted according to whether they shared the same direction of associations: red (same direction) or green (opposing direction).The list of full names of the abbreviated metabolic variables can be found in Table S2.

In meta-analysis across individual cohorts and follow-ups, we identified large number of metabolic variables (*N* = 89) tested to be significantly associated with CA after correcting for FDR at *q* < 0.05 (Figure 2a). For example, albumin, histidine (His), leucine (Leu), phospholipids in small HDL (S_HDL_PL), and diameter for VLDL particles (VLDL_size) were found to decrease with higher CA; whilst citrate, glucose, creatinine, β-hydroxybutyrate (bOHbutyrate), docosahexaenoic acid (DHA), omega-3 fatty acids, glutamine (Gln), tyrosine (Tyr), phenylalanine (Phe), total free cholesterol (Total_FC), and sphingomyelins were among those found to increase with CA the most. (Figure 2a, Table S3).

To test for consistency in response between CA and metabolic variables, we performed additional metabolome-wide association studies stratifying by age groups, additionally adjusting for cohort (Figure 2b**)**. Metabolic variables showing consistent and positive associations with CA across age groups included triglycerides (TG) in IDL, TG variables in four LDL subfractions, and cholesterols in very large HDL particles (XL_HDL_C and XL_HDL_FC). Conversely, VLDL_size, albumin, and lactate were found to be consistently and negatively associated with CA. Although positively associated with CA through meta-analyses, citrate, omega-3, polyunsaturated fatty acids (PUFA), Apolipoprotein B (ApoB), and many cholesterols/cholesterol esters and lipoprotein subfraction measurements showed heterogenous associations with CA across different age ranges. Whereas the increase in citrate levels with age appeared to be driven by older populations, the increases in many cholesterols/cholesterol esters and lipoprotein subfraction measurements appeared to be more prominent in those aged <60 years (Figure 2b, Table S4).

As lifespan may be considered the most relevant phenotypic end-point for studying aging, we examined metabolite associations with time to all-cause mortality in three cohorts (UKCTOCS, SABRE, and WHII) in which mortality data were available, consisting of 10,648 individuals of whom 2312 died during subsequent follow-up. Cohort-specific Cox proportional hazards regression models were adjusted for age, sex, BMI, and ethnicity, and fixed-effect meta-analysis was performed to pool together individual cohort effect estimates (Figure S5, Figure 2c). Seventeen metabolic markers were found positively associated with all-cause mortality after adjusting for false discovery rate (*q* < 0.05), which include Phe, glycoprotein acetyls (GlycA), lactate, bOHbutyrate, acetate, creatinine, glucose, monounsaturated fatty acids (MUFA), triglycerides in seven different lipoprotein subfractions and free cholesterol in small HDL. Forty-nine metabolic biomarkers were negatively associated with all-cause mortality, and PUFA, omega-6, omega-3 fatty acids, and cholesterols and cholesterol esters in IDL were found to be most negatively associated with mortality in our study sample (Figure S5, Table S5). Our biomarker-mortality associations study results are in good agreement with results reported by Deelen et al. (2019), with coefficients of mortality associations of individual metabolites of our analysis strongly correlated to the results reported (Figure S6). Next, we examined correspondence between metabolites associated with age, and those associated with mortality in our dataset (Figure 2c), and observed that while some age-related metabolic changes (e.g., creatinine, Phe, and TG) contribute to mortality risk, at least some metabolites positively associated with CA may in fact be offering a protective effect against premature mortality (e.g., PUFA, omega-3/ omega-6 fatty acids, DHA, and cholesterol esters in IDL).

### 3.2 Multivariable modeling of metabolomic aging

**Figure 3:**
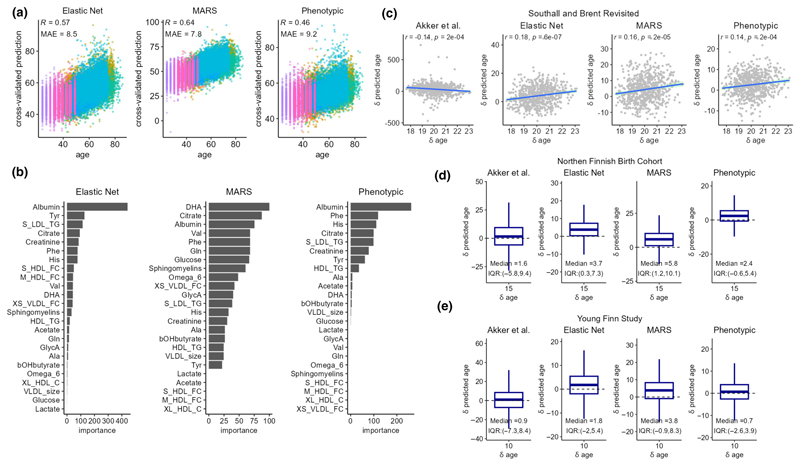
(a) Scatter plots of 7-fold cross-validated predicted age against chronological age. Samples were colored by cohorts. (b) Variable importance (VIP) scores were estimated in the training samples based on the relative importance of predictors in the models (c–e) Longitudinal model predictions of changes in chronological age in Southall and Brent Revisited (SABRE), (d) 1966 Northern Finland Birth Cohort (NFBC1966), (e) Young Finns Study (YFS). Changes in the predicted age was plotted against changes in chronological age at follow-up visits in SABRE, and the boxplot shows the distribution of changes in predicted age during the 15 years and 10 years intervals between follow-up visits in NFBC1966 and YFS.

Multivariable predictors for CA were trained using machine learning approaches including elastic net regression and MARS, using 24 of the most reliable and independent metabolic variables (Figure 3a). Additionally, we also trained a modified elastic net model on CA, which we refer to as “phenotypical aging”, by specifying differential model input weights based on their directionality and strength of their associations with mortality in our study samples. These three models were evaluated using 7-fold cross validations (CV) and leave-one-cohort-out validations (LOCO). Albumin and citrate were estimated to be among the most important predictors in all three CA models (Figure 3b). The overall Pearson’s correlation coefficients (*r*) between CA and the CV predicted age were 0.57, 0.65, and 0.47, and the correlations (*r*) with the LOCO predictions were 0.38, 0.37, and 0.23, respectively, for the elastic net, MARS, and phenotypic age models. (Figure S7). The published Akker et al. CA model performed relatively poorly in our study data, giving a Pearson’s *r* = 0.26 with CA and a mean absolute error (MAE) of around 18 years of age. Among the SABRE, NFBC1966, and YFS cohorts that included repeat metabolomic data, we compared change in predicted age (δ predicted age) with change in CA (δ CA, i.e., years between assessments). We observed significant positive correlation between δ predicted age and δ CA in SABRE for all metabolomic age measures, except the Akker et al. model (Figure 3c) and general increases in median metabolomic age between follow-ups for NFBC1966 (Figure 3d) and YFS (Figure 3e). However, the models generally underpredicted δ metabolomic age relative to δ CA.

### 3.3 Metabolomic aging and age-related phenotypes

**Figure 4:**
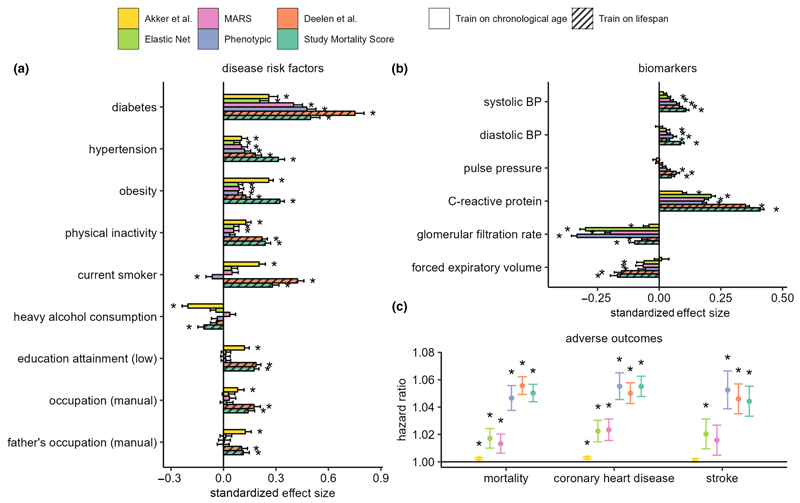
(a) Associations with noncommunicable disease risk factors. Estimates represent standard deviation change in metabolomic age associated with exposure which have been categorized into binary variables. (b) Associations with age-related biomarkers. Estimates represent standard deviation (SD) change in metabolomic age associated with 1 SD unit change in biomarker levels. To avoid individuals from being accounted for more than once in the analysis, samples from YFS2001 and YFS2007, NFBC1966 (31 years), and SABRE2 were excluded in the disease risk factor analysis, and subsequently up to 28,000 samples were included. (c) Associations of metabolomic age models with adverse incident health events. Cox proportional regression models were adjusted for chronological age, sex, and ethnicity, and hazard ratios were estimated per unit of change in metabolomic age. 969, 638 and 715 deaths, respectively, in UKCTOCS, SABRE, and WHII, and 1273 and 442 coronary heart disease events, and 707 and 181 stroke events were, respectively, recorded in the UKCTOCS and SABRE cohorts during the subsequent follow-up period of up to 25 years. Analyses in (a–c) were based on cohort fixed effect inverse variance weighted meta-analyses and linear regression models adjusted for chronological age, sex, and ethnicity. * denotes a *p* < 0.001 and error bars represent the lower and upper limits of the 95% confidence intervals.

Next, we assessed and compared associations of the four metabolomic aging models (trained on CA) and two models trained directly on mortality (Deelen et al. model and a new study mortality score), against noncommunicable disease risk factors and six common biomarkers of aging phenotypes, in analyses adjusted for CA, sex, and ethnicity. Among the risk factors, diabetes, hypertension, and obesity statuses were positively associated (*p* < 0.001) with all metabolomic models, and physical inactivity was also positively associated with five of the six metabolomic scores examined (Figure 4a). Additionally, current smoking status and indicators of lower socioeconomic positions (low education attainment and manual occupation status) were also positively associated with the Akker et al. and Deelen et al. models and the study mortality score. All metabolomic models examined were found positively associated (*p* < 0.001) with CRP (inflammation) and negatively associated with glomerular filtration rate (kidney function). Except for the Akker et al. model, all models were positively associated with SBP and DBP, and negatively associated with forced expiratory volume (Figure 4b).

Furthermore, we investigated the relationship of metabolomic age with incident health events in the cohorts with available data using Cox proportional regression models adjusted for CA, sex, and ethnicity, and fixed effect meta-analysis to combine individual cohort estimates. All metabolomic aging models trained on CA and lifespan were significantly associated with all-cause mortality (*N*_*event*_= 2312) and coronary heart disease (CHD) incidences (*N*_*event*_ = 1715), and with the exception of the Akker et al. model and MARS, all other models were significantly associated (*p* < 0.001) with incidence of stroke (*N*_*event*_ = 888, Figure 4c) Phenotypic aging and the two models of lifespan, Deelen et al. and study mortality score, were most strongly associated with adverse health outcomes, with Hazard Ratios (HRs) for all-cause mortality of 1.047 (95% Confidence Interval (CI): 1.038–1.056), 1.056 (95% CI: 1.049–1.062), and 1.05 (95% CI: 1.044–1.057), respectively, per year of metabolomic age.

To understand the contribution of adiposity to the observed associations with metabolomic age markers, we additionally adjusted for BMI in sensitivity analysis. Associations with hypertension and blood pressure were attenuated for the CA trained model associations. However, adjusting for BMI did not significantly affect study models associations with adverse health outcomes (Figure S8).

### 3.4 Independent assessment in the UK biobank

**Figure 5:**
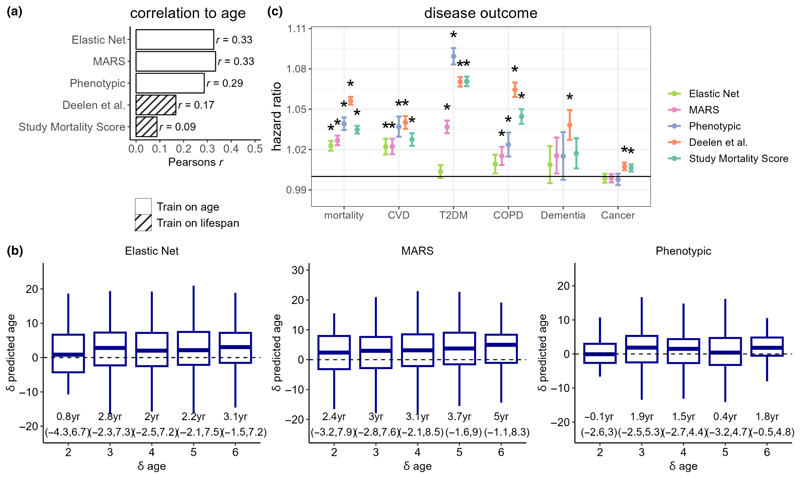
(a) Assessment of metabolomic aging model scores in UK Biobank (*N*_total_ = 101,524). Pearson’s correlation coefficients with chronological age are shown. (b) Longitudinal assessment of metabolomic aging model scores in UK Biobank. (c) Associations of chronological age-adjusted metabolomic age scores with adverse incident events in the UK Biobank. Cox proportional regression models were adjusted for sex and chronological age. Hazard ratios were estimated based on per year of metabolomic age. * denotes a *p* < 0.001 and error bars represent the lower and upper limits of the 95% confidence intervals.

Performance of the metabolomic aging models were tested in the large independent UK Biobank sample (Sudlow et al., 2015), comprising of metabolomic data from 101,524 individuals and accompanied by rich phenotypic and follow-up data. Models trained directly on CA provided modest predictive performance in UKB, with Pearson’s *r* with CA of 0.29, 0.33, and 0.33 for phenotypic age, elastic net, and MARS, respectively. In addition, both models of lifespan (Deelen et al. and study mortality score) also showed significant correlations with CA (Pearson’s *r*: 0.09–0.17, *p* < 1 × 10^−10^) in the UKB (Figure 5a).

Among 1108 UKB participants with longitudinal metabolomic data measured at baseline and at clinical follow-up 2–6 years later, we compared change in metabolomic age (δ metabolomic age) with change in CA (δ CA) for the CA trained models, categorized by years of follow-up. For most follow-up categories, we observed an increase in median metabolomic age over follow-up, except for phenotypic age among those only followed-up for 2 years. The MARS model showed the greatest concordance between δ CA and δ metabolomic age, with a median δ metabolomic age of 5 years (IQR: −1.1–8.3 year) among those with δ CA of 6 years (Figure 5b).

Using Cox proportional regression models adjusted for CA and sex, we studied associations of metabolomic aging models with all-cause mortality (*N*_*event*_ = 6645), CVD (*N*_*event*_ = 2585), T2DM (*N*_*event*_ = 3850), cancer (*N*_*event*_ = 8192), dementia (*N*_*event*_ = 450), and COPD (*N*_*event*_ = 1814) incidences in the UKB samples (Figure 5b, Table S8). All metabolomic aging models tested were significantly associated (*p* < 0.001) with all-cause mortality and CVD. Effect estimates for all-cause mortality ranged from a HR of 1.023 per year of metabolomic age (95% CI: 1.019–1.026) for the Elastic Net model to a HR of 1.056 (95% CI: 1.053–1.059) for the Deelen et al. model. The next best performing model was phenotypic age (HR: 1.039 [95% CI: 1.034–1.039]), which outperformed the MARS model (HR: 1.027 [95% CI: 1.023–1.030]), which in turn outperformed the linear CA trained models for prediction of all-cause mortality. For instance, every additional year of MARS, relative to CA, was associated was a 3% increase in mortality risk. We also stratified our Cox regression analyses by age bands to examine whether associations with all-cause mortality differed among age bands. Generally, associations were stronger in the youngest age band: for those aged under 55 years, each additional year of MARS age was associated with a 4% increase in mortality risk, compared to a 2% increase among those aged over 65 years (Figure S9).

A similar pattern of the relative associations with the metabolomic age models, was observed for CVD. All models except the elastic net model were also found to be significantly associated with incidences of T2DM and COPD. The best performing model for T2DM prediction was phenotypic age (HR: 1.089 [95% CI: 1.083–1.095]), and the Deelen et al. model performed the best for prediction of COPD (HR: 1.064 [95% CI: 1.059–1.070]). Both the mortality score and the Deelen et al. model showed small significant association with cancer incidence, while only the Deelen et al. model was associated with dementia incidence.

## 4 Discussion

In one of the largest epidemiological metabolomic studies to date, we have developed and tested the performance of various multivariable metrics to assess aging as a biological process.

In brief, metabolomics data were generated through NMR spectroscopy in blood serum from nine UK and Finnish cohort studies, covering an age range from 24 to 86 years. We used multivariate adaptive regression splines (MARS) and penalized regression models to predict CA and mortality. Alongside two published metabolomic prediction scores (“Akker et al.” trained on CA, and “Deelen et al.”, trained on all-cause mortality), we examined associations of new CA-adjusted metabolomic age models with aging phenotypes. These metabolomic measures were associated with blood pressure parameters and C-reactive protein levels and inversely associated with glomerular filtration rate. Risk factors associated with age-adjusted metabolomic age scores included obesity, diabetes, smoking, physical inactivity, and low education level. In independent testing in the UK Biobank, correlations with CA were modest, yet all metabolomic model scores predicted all-cause mortality and CVD.

### 4.1 Performance of different models: Prediction of chronological age

One criterion that a biological age estimator should fulfill is that should change with CA (Moskalev, 2019). When we compared our models to the UKB set, correlation with CA was more modest. The MARS model performed the best, based on model fit in the training set and associations with CA and δ CA in the UKB, indicating the value of incorporating nonlinear modeling. However, taken together, models trained on CA provided only moderately improved age prediction performance compared to models trained on lifespan. The models trained on CA in our study also apparently outperformed the previously published Akker et al. model (albeit tested in different independent populations), despite it being trained on a similarly sized dataset. This difference may be due to the additional preprocessing and variable selection steps applied, thereby increasing model stability, and potentially due to use of fasting samples only in our training set (i.e., not in UKB) reducing the influence of recent food intake on metabolite levels. Overall, as predictors of CA across independent test sets, models based on NMR metabolomic data (LOCO cross-validated *r* = 0.23–0.38, and *r* = 0.29–0.33 in UKB) fall a long way short of gold-standard data types such as DNA methylation (Hannum et al., 2013; Horvath, 2013) which frequently show a *r* of greater than 0.9, although models based on NMR metabolomic data perform somewhat similarly to telomere length (Bekaert et al., 2005; Vaiserman & Krasnienkov, 2021).

### 4.2 Prediction of mortality and disease incidence

Biological age estimators should also predict mortality better than CA and predict the early stages of a specific age-related disease (Ferrucci et al., 2020; Levine, 2013). To test this, we assessed the metabolomic scores adjusted for CA, against mortality, and incidence of age-related disease. All models were able to predict mortality in both the training set and the UKB, with generally similar estimates in both populations, with the greatest effect size seen for the Deelen mortality estimator. The Deelen et al. model was also the only model that could predict incidence of all age-related diseases tested (CVD, T2DM, cancer, dementia, and COPD) in UKB suggesting it is able to capture generalizable age-related disease susceptibility. Phenotypic age performed well in terms of mortality and disease prediction, while still offering comparable associations with CA in UKB to the models trained purely on CA. Effects sizes in predicting time-to-death for the presented metabolic models, particularly Deelen et al. and phenotypic aging model, were comparable to those of reported biological age assessments based on clinical markers, such as BioAge and PhenoAge (Kuo et al., 2021), and epigenetic clocks such as the Horvath, Hannum, and DNAm PhenoAge clocks (Hannum et al., 2013; Horvath, 2013; Levine et al., 2018), although smaller than the GrimAge epigenetic clock (Lu et al., 2019). However, the advantage of age models based on NMR metabolomic data compared to other more complex indicators is that a single analysis is required rather than assaying multiple clinical markers and they are relatively cost-effective, especially compared to acquisition of epigenetic data. Also, our study results have shown that whilst models trained on CA were consistently associated with mortality and morbidity after adjustment for sample age, models trained or capturing lifespan information, such as Deelen et al and our study mortality and phenotypic aging scores, will likely show significantly stronger effect against health outcomes.

### 4.3 Physiological interpretation

In meta-analysis, we observed generally consistent decreases with age in metabolic measures including albumin, a marker of liver and kidney function, essential amino acid histidine, the branched-chain amino acid leucine, phospholipids in small HDL, and the diameter of VLDL. Conversely, increases with age were observed in citrate, glucose, amino-acids creatinine and glutamine, aromatic amino acids tyrosine and phenylalanine, the ketone body β-hydroxybutyrate, omega-3 fatty acids, the degree of unsaturation of fatty acids, triglycerides, and large and very large HDL. The increase in triglyceride levels is well-established in aging, as it reflects changes in plasma TG clearance, adipose tissue lipolysis, and the partitioning of fat (Spitler & Davies, 2020). Citrate, in addition to its key role as an energy hub metabolite, may be released through increased bone resorption (Granchi et al., 2019) and has also recently been demonstrated to be an independent marker of extracellular senescence in in-vitro models (James et al., 2018). Also, increased blood level of phenylalanine with age has previously been associated with dysregulated phenylalanine catabolism and cardiac impairment in mice (Czibik et al., 2021), and age-related reduction in creatinine clearance has been a key marker of decline in kidney function (Weinstein & Anderson, 2010). While these associations have generally been previously reported (Panyard et al., 2022), we confirmed their relationship with age in a multicohort setting. Furthermore, we found that metabolic associations with mortality well replicated previously reported findings (Deelen et al., 2019). We found that some of these metabolites were related to mortality in a direction consistent with the relationship with age including creatinine, phenylalanine, and triglycerides, some age-related metabolites had neutral or nonsignificant relationship with mortality, while others particularly DHA, omega-3 fatty acids, and the degree of unsaturation of fatty acids showed inverse relationships with mortality. Given that circulating metabolites have distinct physiological and regulatory functions, we speculate that some metabolites showing different directions of association to mortality and age may in fact be offering a protective/adaptive or neutral response to the physiological aging processes. For instance, DHA is thought to reduce oxidative stress and inflammation by modulating cyclooxygenase, lipoxygenase, and cytochrome P450 lipid mediator activities (Swanson et al., 2012; Zhang et al., 2013).

The models presented trained on CA include metabolites with neutral and potentially adaptive metabolic effects, yet remarkably still provide additional prediction of mortality, suggesting the models are capturing a higher-level picture of metabolic aging, which overall contributes to mortality risk. While aging markers trained on mortality are more sensitive to aging risk factors and show improved prediction of age-related disease generally (Lu et al., 2019), they will to a greater extent capture extrinsic contributions, such as early effects of disease, to metabolic aging. Within this study, we found that metabolic age models were sensitive to classical and modifiable risk factors of mortality (Stringhini et al., 2017), and also related to clinical biomarkers of system function, including blood pressure, C-creative protein, forced expiratory volume, and glomerular filtration rate. Unexpectedly, heavy alcohol use appeared to be negatively associated with some metabolomic age models, which may be related to the effects alcohol consumption has on metabolites such as citrate (Wurtz et al., 2016), illustrating a limitation of the metabolic modeling approach for certain risk factors.

### 4.4 Strengths and limitations

The use of multiple cohorts covering most of adult life is one of the strengths of this study and particularly important for analysis of metabolites, which may be impacted by both endogenous factors such as aging and exogenous factors such as diet, since the relationship between age and exogenous factors (cohort effects) will likely be stronger within single cohorts. The use of some repeat samples, although in limited number, also increases the ability to detect endogenous aging effects, while the use of fasting samples in our training set has lessened the possible influence of diet. However, the comparison cohort, UKB, is based on nonfasting EDTA-plasma samples, which may explain some of the relatively poor replication of the association with age in UKB. Another important limitation is that this study was mainly based on cross-sectional data, which is more susceptible to cohort effects than studies based on longitudinal data and does not allow assessment of trajectories of aging over time (Ala-Korpela et al., 2023). Furthermore, the use of only Northern European cohorts may limit generalizability to other populations. Nevertheless, the main strength was the use of large, independent training and test sets, allowing completely unbiased assessment of model performance.

Metabolic profiling based on NMR provides both strengths and limitations for development of ageing metrics in a multicohort setting. The main advantage is that it is inherently quantitative, enabling comparable analyses of datasets across cohorts, and captures both small molecules and lipid metabolites. It is also high-throughput and cost-effective allowing the large population samples required for precise estimation of age-associations. The main limitation is the lower coverage of NMR compared to mass-spectrometry based methods, meaning that only the most abundant metabolites are detected, and many important and specific age-related metabolites may be missed. Identified metabolites such as citrate and DHA are undoubtedly important components of the aging metabolome, being both among those most strongly associated with age in previous studies that employed broader MS-based analysis (Darst et al., 2019; Menni et al., 2013). However, other key aging metabolites such as steroids, acylcarnitine, and tryptophan metabolites are not assayed by the current Nightingale NMR platform. Future metabolic aging studies will need to combine broad, highly sensitive metabolomics with careful control of technical variation to allow combination across studies.

## 5 Conclusions

We have developed and tested various metabolic aging metrics in a very large dataset. We found that the Deelen et al. model provides the most consistent prediction of mortality and all age-related diseases tested and is therefore a good candidate model for studies investigating metabolically mediated effects on lifespan. MARS, a nonlinear method was found to improve prediction of CA over other modeling techniques. Our phenotypic aging model directly predicts CA, while also providing good predictive ability of mortality and multiple age-related diseases and presents a good candidate for studies of overall metabolic aging. Although we have shown that NMR metabolomics can only provide moderate prediction of CA across independent test sets, the technique provides valuable information regarding metabolic health, which is intricately linked to population aging. We expect that future studies incorporating broader metabolomic analytical techniques will allow more comprehensive and specific assessment of metabolic aging. These models may have utility for large-scale epidemiological analysis, allowing assessment of aging risk factors and mechanisms and stratification and identification of at-risk groups.

## Supplementary Material

Additional supporting information can be found online in the Supporting Information section at the end of this article.

Supplementary Material

## Data Availability

The data that support the findings of this study are available upon application to steering committee of each cohort. Multivariable metabolomic models of biological age generated in this study are available in the supplemental material of this article and via GitHub: https://github.com/chungholau/NMR-metabolomic-age
