# Supplementary material for: NMR metabolomic modeling of age and lifespan: A multicohort analysis
Source: Aging Cell. Author manuscript; Available in PMC 2024 Jul 22. (PMC11258446; doi:10.1111/acel.14164)
Supplement: Supplementary Material [file EMS196749-supplement-Supplementary_Material.zip › acel14164-sup-0001-appendixs1.docx]

# Supplementary Materials

**Ageing models**

**24 pruned metabolite variable set**: Lactate, GlycA, Creatinine, Albumin, Tyr, Val, Phe, His, Gln, Ala, bOHbutyrate, Acetate, Glucose, Citrate, Omega_6, DHA, Sphingomyelins, HDL_TG, VLDL_size, S_HDL_FC, M_HDL_FC, XL_HDL_C, S_LDL_TG, XS_VLDL_FC

**Study mortality score (mortality, standardised values)**: S_LDL_TG × 0.01168 + Omega_6 × (-0.10244) + Glucose × 0.00103 + His × (-0.07158) + Phe × 0.14083 + Val × (-0.01078) + GlycA × 0.07416

**Deelen model (relative mortality, standardised values)**: XXL_VLDL_L × log (0.80) + S_HDL_L × log (0.87) +VLDL_size × log (0.85) +PUFA_FA × log (0.78) +Glucose × log (1.16) +Lactate × log (1.06) +His × log (0.93) +Ile × log (1.23) +Leu × log (0.82) +Val × log (0.87) +Phe × log(1.13) +Acetoacetate × log(1.08) +Albumin × log(0.89) + GlycA × log(1.32)

**Akker model (absolute values)**:

58.62 +

( ( ( Acetoacetate - 0.04319 ) / 0.03465 ) × 1.056 ) +

( ( ( Acetate - 0.0445 ) / 0.01919 ) × 0.6887 ) +

( ( ( Ala - 0.3006 ) / 0.0767 ) × -0.3769 ) +

( ( ( Albumin - 0.08774) / 0.006863 ) × -2.843 ) +

( ( ( APOA1 - 1.594 ) / 0.2002 ) × 0.9177 ) +

( ( ( APOB - 0.9732 ) / 0.2213 ) × 8.111 ) +

( ( ( Citrate - 0.09357 ) / 0.02787 ) × 2.501 ) +

( ( ( Creatinine - 0.07223 ) / 0.01835 ) × 2.168 ) +

( ( ( DHA - 0.1447 ) / 0.05478 ) × -0.8051 ) +

( ( ( Omega_3 - 0.4114 ) / 0.134 ) × 7.364 ) +

( ( ( FAW3_FA - 3.576 ) / 0.9525 ) × 2.75 ) +

( ( ( Omega_6 - 3.871 ) / 0.7695 ) × 63.88 ) +

( ( ( FAW6_FA - 33.76 ) / 3.567 ) × -10.21 ) +

( ( ( Glucose - 4.8 ) / 1.611 ) × 1.394 ) +

( ( ( Gln - 0.4528 ) / 0.07966 ) × 3.844 ) +

( ( ( GlycA - 1.359 ) / 0.2039 ) × 0.1866 ) +

( ( ( HDL2_C - 0.8999 ) / 0.3046 ) × -161.3 ) +

( ( ( HDL3_C - 0.4698 ) / 0.06615 ) × -35.57 ) +

( ( ( HDL_C - 1.37 ) / 0.3277 ) × 187.4 ) +

( ( ( HDL_size- 9.972 ) / 0.2498 ) × 1.254 ) +

( ( ( His - 0.05925 ) / 0.01482 ) × -2.084 ) +

( ( ( IDL_C - 0.6855 ) / 0.1948 ) × -0.04409 ) +

( ( ( IDL_L - 1.069 ) / 0.2802 ) × -3.969 ) +

( ( ( Ile - 0.05285 ) / 0.02023 ) × -1.844 ) +

( ( ( L_LDL_L - 1.171 ) / 0.3441 ) × -23 ) +

( ( ( LA - 3.099 ) / 0.6966 ) × -3.273 ) +

( ( ( Lactate - 1.232 ) / 1.032 ) × 1.868 ) +

( ( ( LDL_C - 1.488 ) / 0.5062 ) × 15.22 ) +

( ( ( LDL_size - 23.65 ) / 0.119 ) × 0.2465 ) +

( ( ( Leu - 0.06167 ) / 0.01617 ) × -4.118 ) +

( ( ( M_HDL_L - 0.8082 ) / 0.1624 ) × -5.544 ) +

( ( ( M_LDL_L - 0.6606 ) / 0.2066 ) × 33.28 ) +

( ( ( M_VLDL_L - 0.6664 ) / 0.3765 ) × -6.233 ) +

( ( ( MUFA - 2.904 ) / 0.9051 ) × -8.85 ) +

( ( ( MUFA_FA - 24.83 ) / 3.645 ) × -68.24 ) +

( ( ( Phosphatidylc - 1.985 ) / 0.3749 ) × -3.59 ) +

( ( ( Phe - 0.04339 ) / 0.008491 ) × 2.939 ) +

( ( ( PUFA - 4.282 ) / 0.8429 ) × -67.3 ) +

( ( ( PUFA_FA - 37.34 ) / 3.682 ) × -61.45 ) +

( ( ( S_HDL_L - 1.004 ) / 0.1017 ) × 4.017 ) +

( ( ( S_LDL_L - 0.4282 ) / 0.1244 ) × -10.42 ) +

( ( ( S_VLDL_L - 0.7053 ) / 0.2318 ) × -9.983 ) +

( ( ( Total_C - 4.42 ) / 0.9903 ) × -28.22 ) +

( ( ( Total_TG- 1.397 ) / 0.6807 ) × 4.872 ) +

( ( ( SFA - 4.375 ) / 0.9667 ) × -19.06 ) +

( ( ( SFA_FA - 37.84 ) / 1.867 ) × -31.47 ) +

( ( ( Sphingomyelins - 0.4548 ) / 0.08845 ) × 1.705 ) +

( ( ( Cholines - 2.293 ) / 0.3814 ) × -3.358 ) +

( ( ( Total_FA - 11.56 ) / 2.482 ) × 23.67 ) +

( ( ( Phosphoglyc - 1.898 ) / 0.3669 ) × 5.78 ) +

( ( ( Tyr - 0.06123 ) / 0.01556 ) × 2.209 ) +

( ( ( UNSAT - 1.215 ) / 0.0745 ) × -0.49 ) +

( ( ( Val - 0.1574 ) / 0.03815 ) × 1.656 ) +

( ( ( VLDL_C - 0.8767 ) / 0.2799 ) × 15.42 ) +

( ( ( VLDL_size- 36.79 ) / 1.361 ) × 3.839 ) +

( ( ( XS_VLDL_L - 0.5663 ) / 0.1263 ) × 5.976 )

**Cohort information**

*Avon Longitudinal Study of Parents and Children*

The Avon Longitudinal Study of Children and Parents (ALSPAC) was established to understand how genetic and environmental characteristics influence health and development in parents and children. All pregnant women resident in Avon, UK with expected dates of delivery 1st April 1991 to 31st December 1992 were invited to take part in the study. Of the original 14,541 initial pregnancies, 338 were from a woman who had already enrolled with a previous pregnancy, meaning 14,203 unique mothers were initially enrolled in the study. As a result of the additional phases of recruitment, a further 630 women who did not enroll originally have provided data since their child was 7 years of age. This provides a total of 14,833 unique women (G0 mothers) enrolled in ALSPAC as of September 2021. G0 partners were invited to complete questionnaires by the mothers at the start of the study and they were not formally enrolled at that time. 12,113 G0 partners have been in contact with the study by providing data and/or formally enrolling when this started in 2010. 3,807 G0 partners are currently enrolled. Study data were collected and managed using REDCap electronic data capture tools hosted at the University of Bristol (Harris et al., 2009). REDCap (Research Electronic Data Capture) is a secure, web-based software platform designed to support data capture for research studies. Consent for biological samples has been collected in accordance with the Human Tissue Act (2004) and ethical approval for the ALSPAC study was obtained from the ALSPAC Ethics and Law Committee and the Local Research Ethics Committees. Informed consent for the use of data collected via questionnaires and clinics was obtained from participants following the recommendations of the ALSPAC Ethics and Law Committee at the time. Please note that the study website contains details of all the data that is available through a fully searchable data dictionary and variable search tool: <http://www.bristol.ac.uk/alspac/researchers/our-data/>

*Northern Finnish Birth Cohort 1966*

The study was started in the two Northernmost provinces in Finland (Oulu and Lapland) in the year 1965 when the mothers were pregnant. Data on the individuals born into this cohort was collected since the 16th gestational week as well as their mothers and, to a lesser extent, fathers. The cohort included 12,055 mothers and they had 12,068 deliveries (13 women delivered twice). Cases belonging to survey were determined by the calculated term. A small percentage of the births occurred towards the end of 1965 and early in 1967. The calculated term, as was customary at that time, was counted from the first day of the last menstrual period. Where this date was unknown the expected term was estimated from the date of commencement of foetal movements and progress of the pregnancy. The study covered all live born and stillborn infants with birth weight of 600 grams or more. According to the Finland's central Office of Statistics, births in the study area during 1966 totaled 12,527, so study population comprised 96.3% of all births during 1966 in that area. Altogether 12,231 children were born into the cohort, 12,058 of them live-born. The original data have been supplemented by data collected with postal questionnaires at the ages of 1, 14, 31 and 48 years and various hospital records and national register data.

*Young Finns Study*

The program was launched in Finland in the late 1970’s to study cardiovascular risk in the youth. The multi-centre study, called The Cardiovascular Risk in Young Finns, was designed to study the risk factors and precursors of cardiovascular diseases and their determinants in children and adolescents. Two pilot studies were carried out in 1978 and 1979, and the first cross-sectional study in 1980. Thereafter, this cohort has been followed-up several times, and the latest field study was conducted in 2011/12. The first cross-sectional survey was conducted in 1980. Total sample size was 4,320 boys and girls in 6 age cohorts (aged 3, 6, 9, 12, 15 and 18). These subjects were randomly chosen from the national register. A total of 3,596 subjects (83.2% of those invited) participated in the study in 1980. After that, several follow-up studies of this cohort have been conducted. The participation rates in the follow-up studies have varied between 60 and 80%. In the latest follow-up in 2011/12 a total of 2,063 subjects were examined (57% of the original cohort).

*MRC National Survey of Health and Development*

The NSHD has informed UK health care, education and social policy for more than 50 years and is the oldest and longest running of the British birth cohort studies. Today, with study members in their seventies, the NSHD offers a unique opportunity to explore the long-term biological and social processes of ageing and how ageing is affected by factors acting across the whole of life. From an initial maternity survey of 13,687 of all births recorded in England, Scotland and Wales during one week of March 1946, a socially stratified sample of 5,362 singleton babies born to married parents was selected for follow-up. This sample comprises the NSHD cohort and participants have been studied 24 times. During their childhood, the main aim of the NSHD was to investigate how the environment at home and at school affected physical and mental development and educational attainment. During adulthood, the main aim was to investigate how childhood health and development and lifetime social circumstances affected their adult health and function and how these change with age. Now, as participants pass retirement age, the research team is developing the NSHD into a life course study of ageing.

*Southall And Brent REvisited Study*

The Southall And Brent REvisited Study (SABRE) is the largest tri-ethnic population-based cohort in the UK, involving nearly 5,000 European, Indian Asian and African Caribbean men and women. It investigates the causes of diabetes and disorders of the heart and circulation. The participants were aged 40-69 when first studied between 1989 and 1991. In 2008 – 2011 a comprehensive combined morbidity and mortality follow up was carried out, together with non-invasive clinical measurements in order to quantify sub-clinical disease. SABRE visit 2 tested hypotheses generated from the Southall and Brent baseline studies and ongoing mortality follow-up. SABRE Visit 3 (25-year follow-up visit) started in July 2014 and will collect data on participants and their partners. The aims of the study are to build on what has been learned from the first study. Changes in the health of the heart and circulation will be measured, with a special focus on the health of the blood vessels of the brain, as well as early signs of diabetes. The study will also look at physical function and how well (or otherwise) people are keeping as they get older.

*Whitehall II Study*

The Whitehall II study was established to investigate the causes of social inequalities in health (Marmot & Brunner, 2005). A cohort of 10,308 participants aged 35-55, of whom 3,413 were women and 6,895 men, was recruited from the British Civil Service in 1985. Since this first wave of data collection, self-completion questionnaires and clinical data have been collected from the cohort every two to five years with a high level of participation. The Whitehall II study has shown the importance of psychosocial factors such as work stress and work-family conflict in heart disease and diabetes. These are in addition to the contribution of unhealthy behaviours and traditional risk factors (such as high blood pressure).

*Caerphilly Prospective Study*

The Caerphilly Prospective Study (CAPS) was set up by the MRC Epidemiology Unit (South Wales). At that time, it was the fifth prospective study of cardiovascular disease in the United Kingdom, although only the second population-based study, after the British Regional Heart Study. Its initial aims were to examine the importance of lipids, haemostatic factors, and hormones such as testosterone, cortisol and insulin (Lichtenstein et al 1987) in the development of ischaemic heart disease (IHD). Subsequently, other hypotheses were included with a specific interest in platelet function, and psychosocial variables. With the ageing of the cohort, additional outcomes have been included in particular stroke, hearing problems and cognitive function. The initial design attempted to contact all men aged 45 to 59 years from the town of Caerphilly and adjoining villages. Two thousand, five hundred and twelve subjects (response rate 89%) identified from the electoral register and general practice lists were examined between July 1979 until September 1983 (phase I). Men were initially seen at an evening clinic, where they completed a questionnaire, had anthropometric measures and an ECG taken. They also completed a food frequency questionnaire at home (Fehily et al 1994). They subsequently re-attended an early morning clinic to have fasting blood samples for a wide variety of tests. Quality control was examined by the use of both "blind" split samples as well as a second repeat measure on a random sub-sample to examine intra-individual variation.

*UK Collaborative Trial of Ovarian Cancer Screening Longitudinal Women’s Cohort*

The cohort is the bioresource built in the course of the United Kingdom Collaborative Trial of Ovarian Cancer Screening (UKCTOCS) (Jacobs et al., 2016). The latter is designed to test the hypothesis that ovarian cancer screening can save lives by detecting the disease earlier. Between April 2001-Sept 2005, 202,638 postmenopausal women, aged 50-74 years were recruited through 13 trial centers in England, Wales and Northern Ireland. Women were randomly allocated to one of three groups (i) control (C) - no screening (ii) multimodal screening (MMS) - annual blood test for serum CA125 measurement. The results were interpreted using the ‘Risk of Ovarian Cancer Algorithm’, with transvaginal ultrasound as a second line test in case of abnormality (iii) ultrasound screening (USS) – annual and second line tests were transvaginal scans. Women in the screen arms underwent a total of 673,765 annual screens till 31st December 2011. The whole cohort is linked to multiple UK electronic health records with ongoing active follow-up.

*British Women’s Heart and Health Study*

The British Women's Heart and Health Study (BWHHS) is a prospective cohort study of cardiovascular disease in women aged over 60 years, in England, Scotland and Wales (Lawlor, Bedford, Taylor, & Ebrahim, 2003). Set up in 1999 to complement the British Regional Heart Study (BRHS), to describe and establish risk factors and the differences in their impact in women compared to the men followed up by the BRHS. The study selected women at random from 24 GP practices, in 23 towns from 1999 to 2000. Of the 7,296 invited, 4,286 (60%) were recruited and attended the baseline examinations and completed questionnaires. Follow-up consisted of postal questionnaires and regular reviews of General Practitioners’ medical records.

*UK Biobank*

UK Biobank (UKB) is a very large and detailed prospective study with over 500,000 UK participants, collected and continues to collect extensive phenotypic and genotypic detail about its participants, including data from questionnaires, physical measures, sample assays, accelerometry, multimodal imaging, genome-wide genotyping and longitudinal follow-up for a wide range of health-related outcomes. Participants were 40–69 years old when recruited in 2006–2010. UK Biobank is available for open access, without the need for collaboration, to any bona fide researcher who wishes to use it to conduct health-related research for the benefit of the public (Sudlow et al., 2015). Incident cases of CVD, COPD and dementia were accessible through algorithms made publicly available by the UKB using data from hospital and death registers: <https://biobank.ndph.ox.ac.uk/showcase/showcase/docs/alg_outcome_stroke.pdf>, <http://biobank.ndph.ox.ac.uk/showcase/showcase/docs/alg_outcome_mi.pdf>, <http://biobank.ndph.ox.ac.uk/showcase/showcase/docs/alg_outcome_dementia.pdf>, <http://biobank.ndph.ox.ac.uk/showcase/showcase/docs/alg_outcome_copd.pdf> . For T2DM incident cases we used the following International Classification of Diseases 10^th^ revision (ICD-10) codes: E11.0, E11.1, E11.2, E11.3, E11.4, E11.5, E11.6, E11.7, E11.8, E11.9, while incident diagnosis of cancer recorded as all cancer (ICD-10: C00-97) including melanoma.

# Supplemental Figures


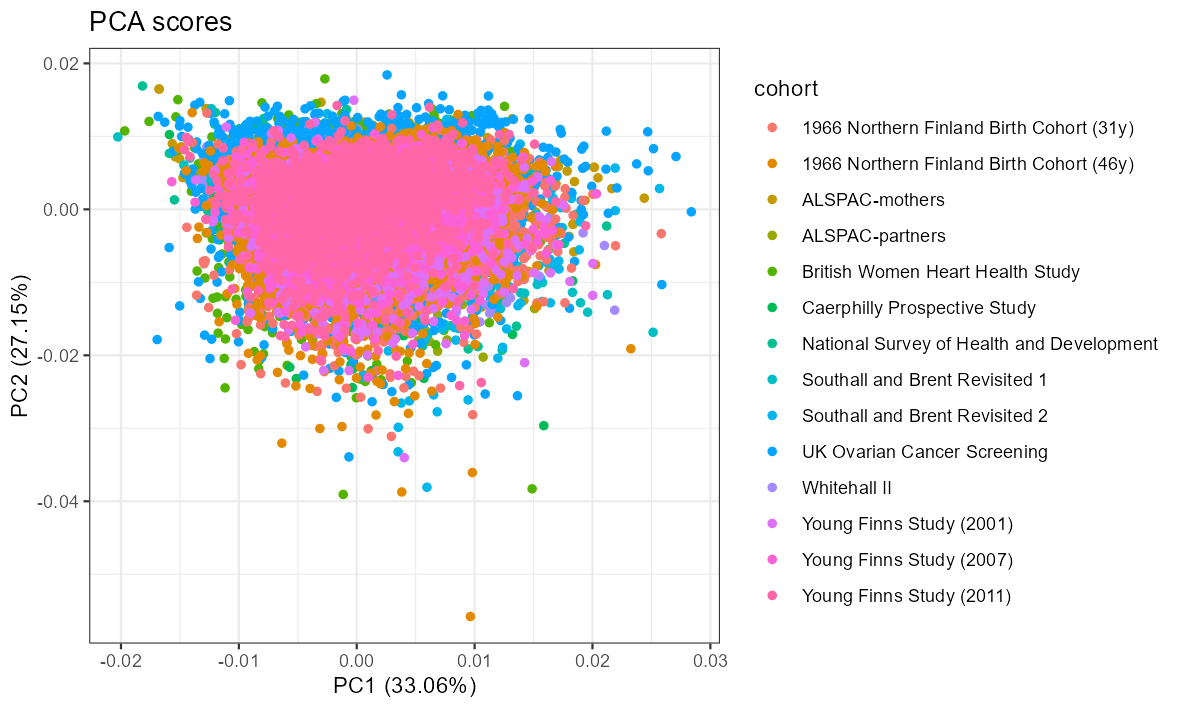


***Supplemental Figure 1*** Principal component analysis score plot. Data were scaled using standard deviation and mean centred prior to PCA analysis.

*
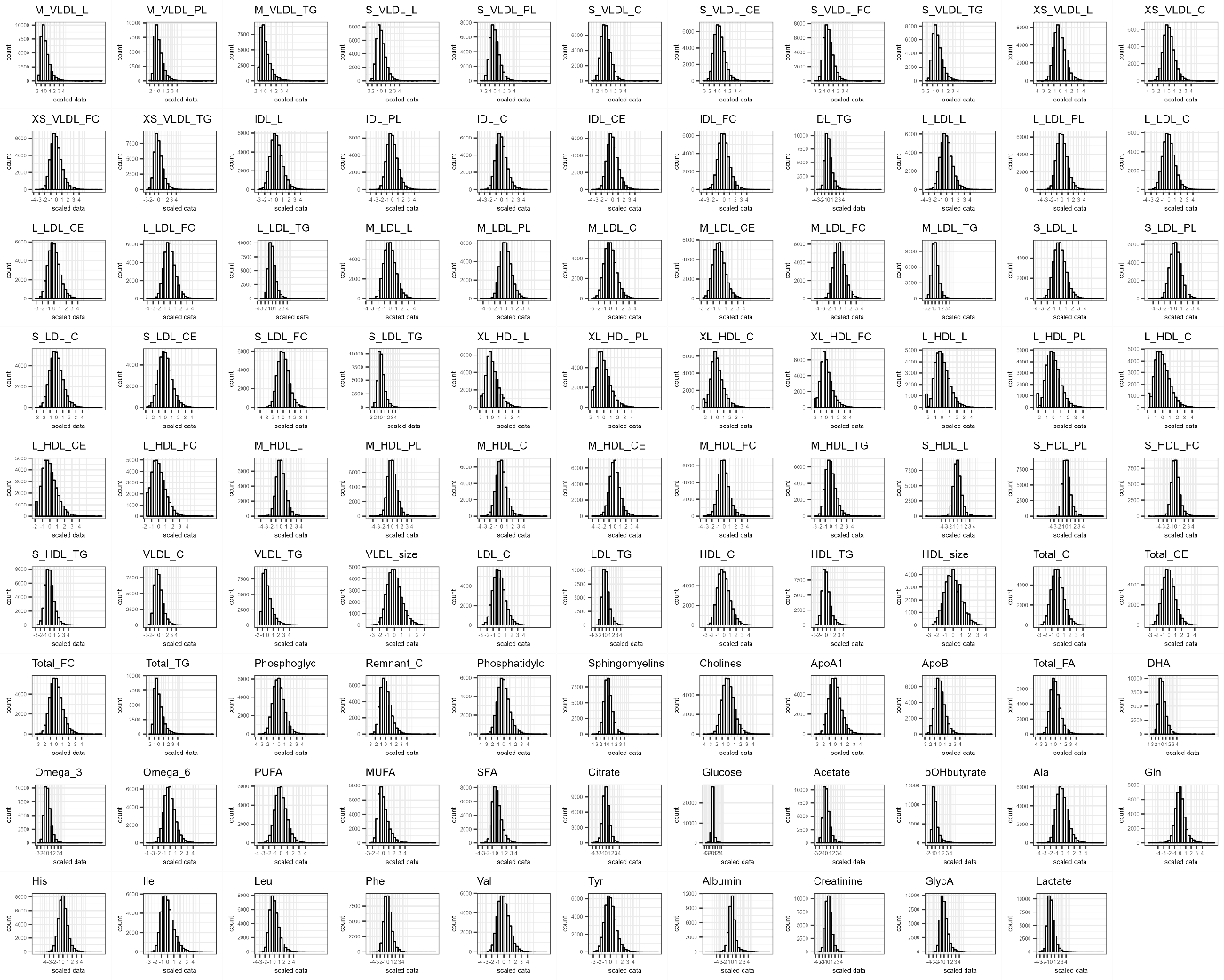
*

***Supplemental Figure 2*** Histograms of the 98 variables included in the study


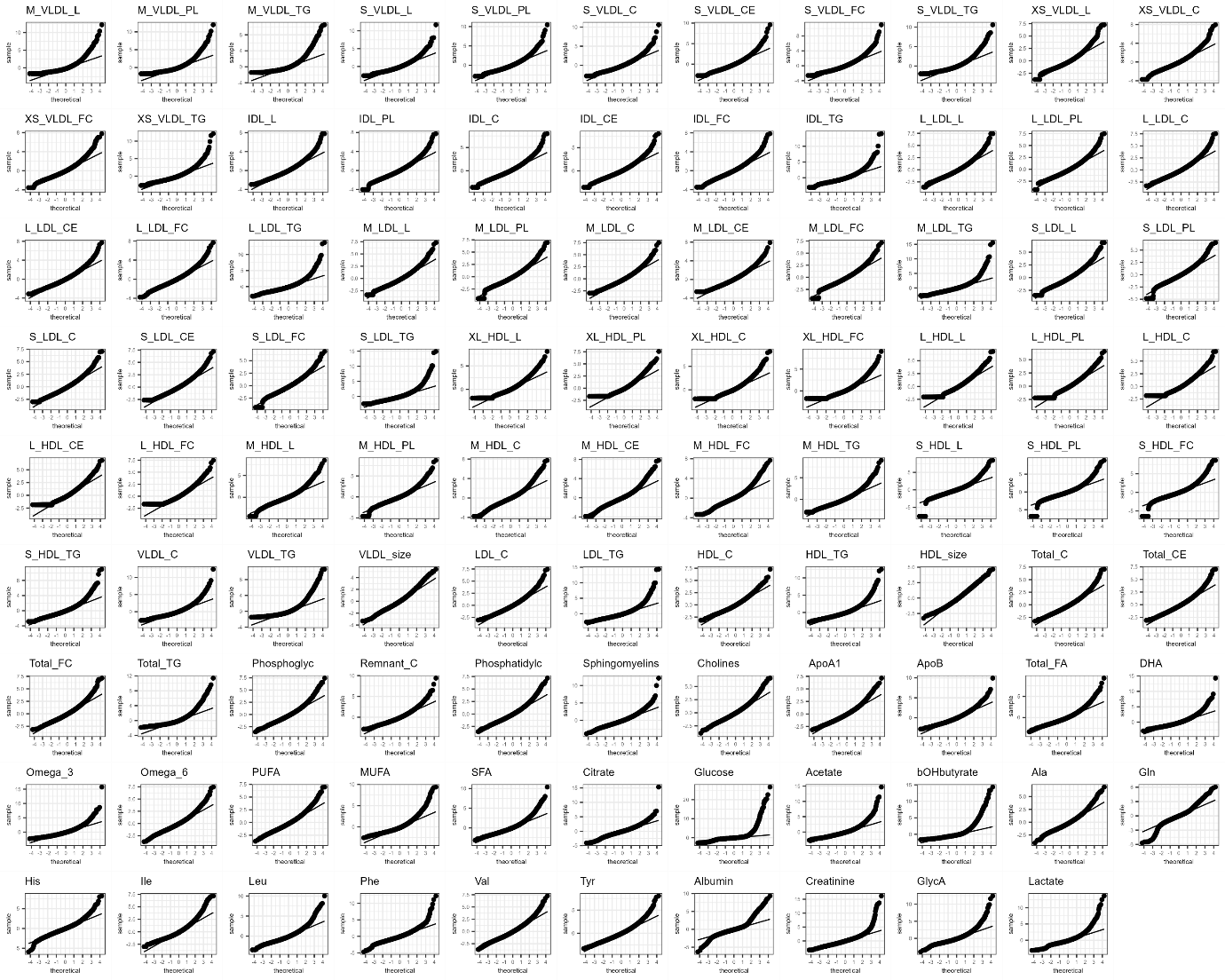


***Supplemental Figure 3*** Quantile-quantile plot of the 98 variables included in the study

**
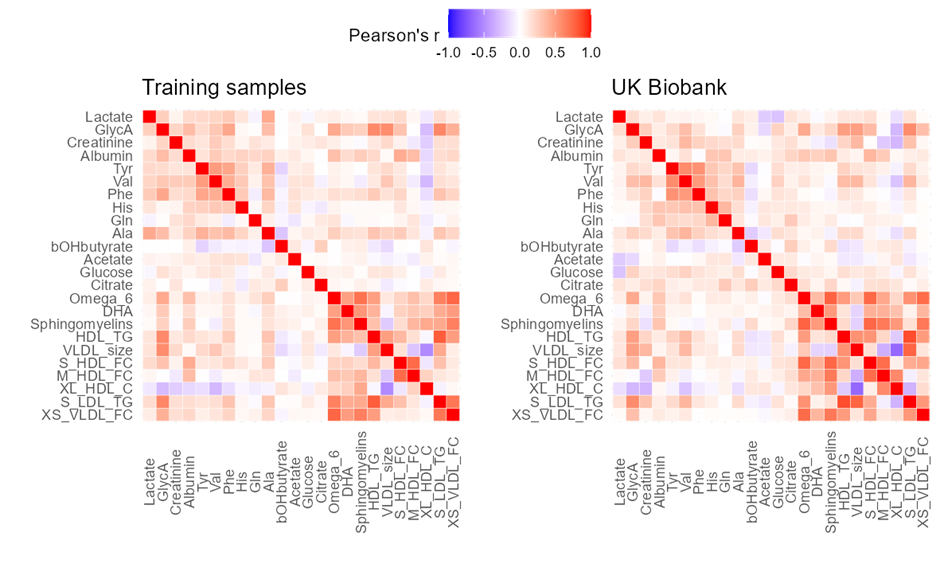
**

***Supplemental Figure 4 metabolic variable pairwise Pearson’s correlations***

**
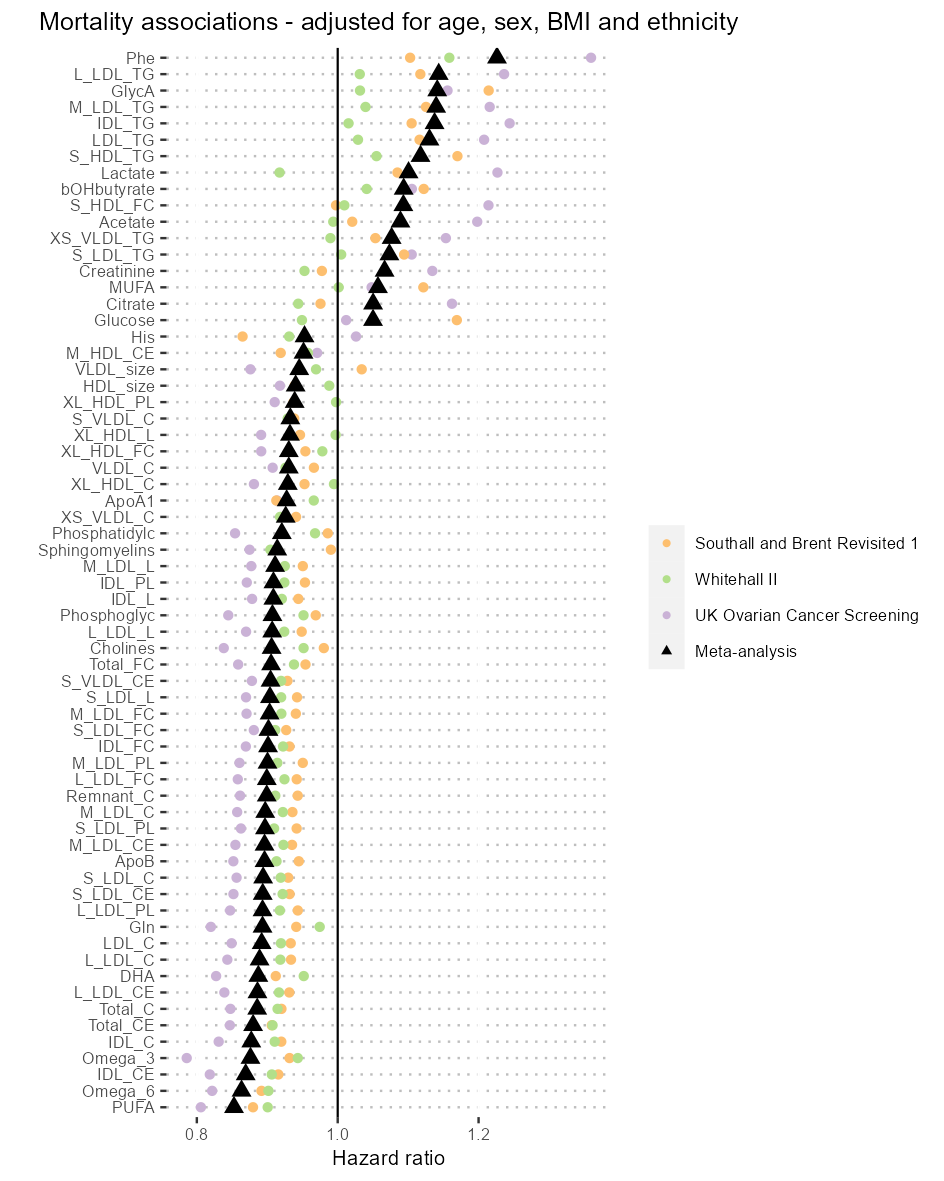
**

**Supplemental Figure 5** *NMR metabolome associations with all-cause mortality in WHII, SABRE, and UKCTOCS. Cohort-specific Cox proportional hazards regression models were adjusted for age, sex, BMI and ethnicity; fixed effected meta-analysis was performed to pool together individual cohort estimates, and significant positive and negative associations with mortality after correcting for false discovery rate (q < 0.05) were shown.*


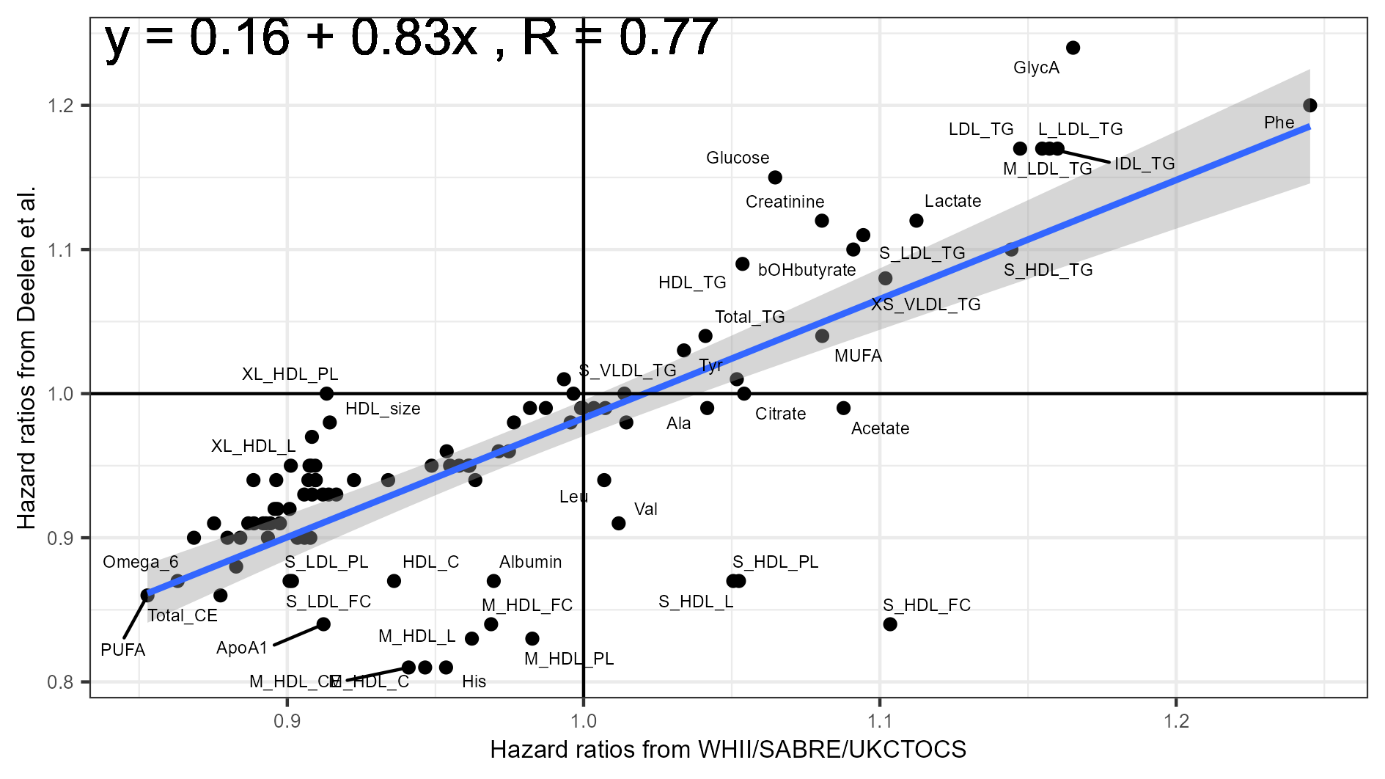


***Supplemental Figure 6*** *Mortality associations compared to published values obtained from Deelen’s et.al. Mortality coefficients values were obtained from Deelen’s published paper (Deelen et al., 2019). “Unadjusted model coefficients” were extracted from Deelen’s paper Supp Data 1. Deelen’s mortality coefficients were adjusted for age and sex and cohort. The estimates represent ln(hazard) per standard deviation change in metabolite level.*


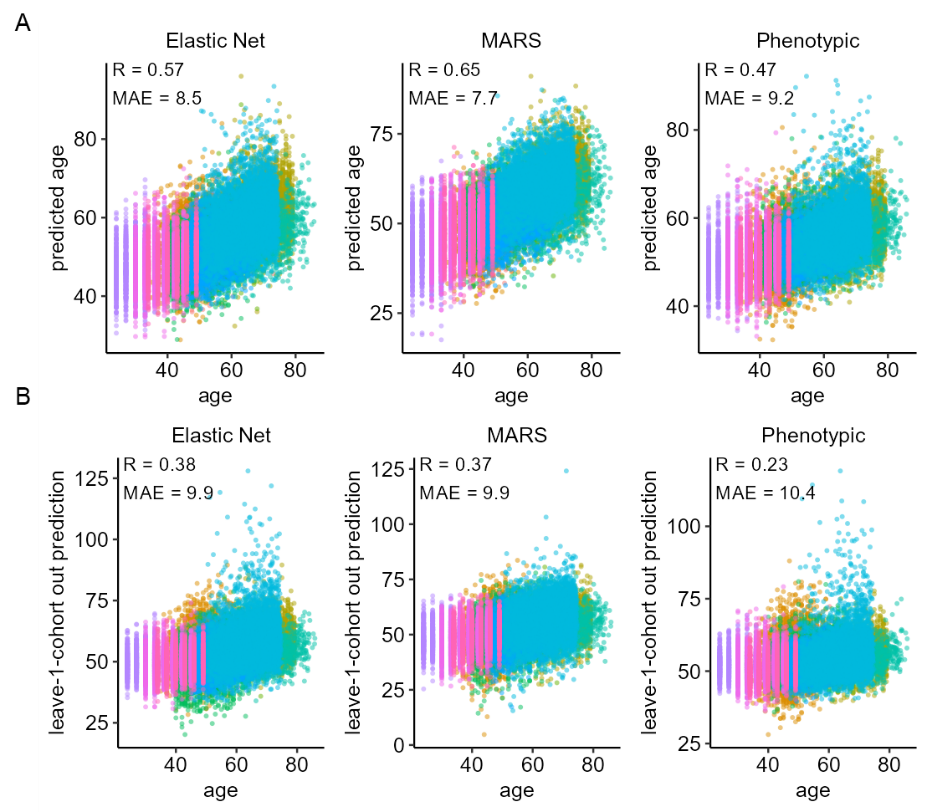


***Supplemental Figure 7*** *Fitted model prediction (top) and leave-one-cohort-out cross validation of modelled age predictions (bottom).*


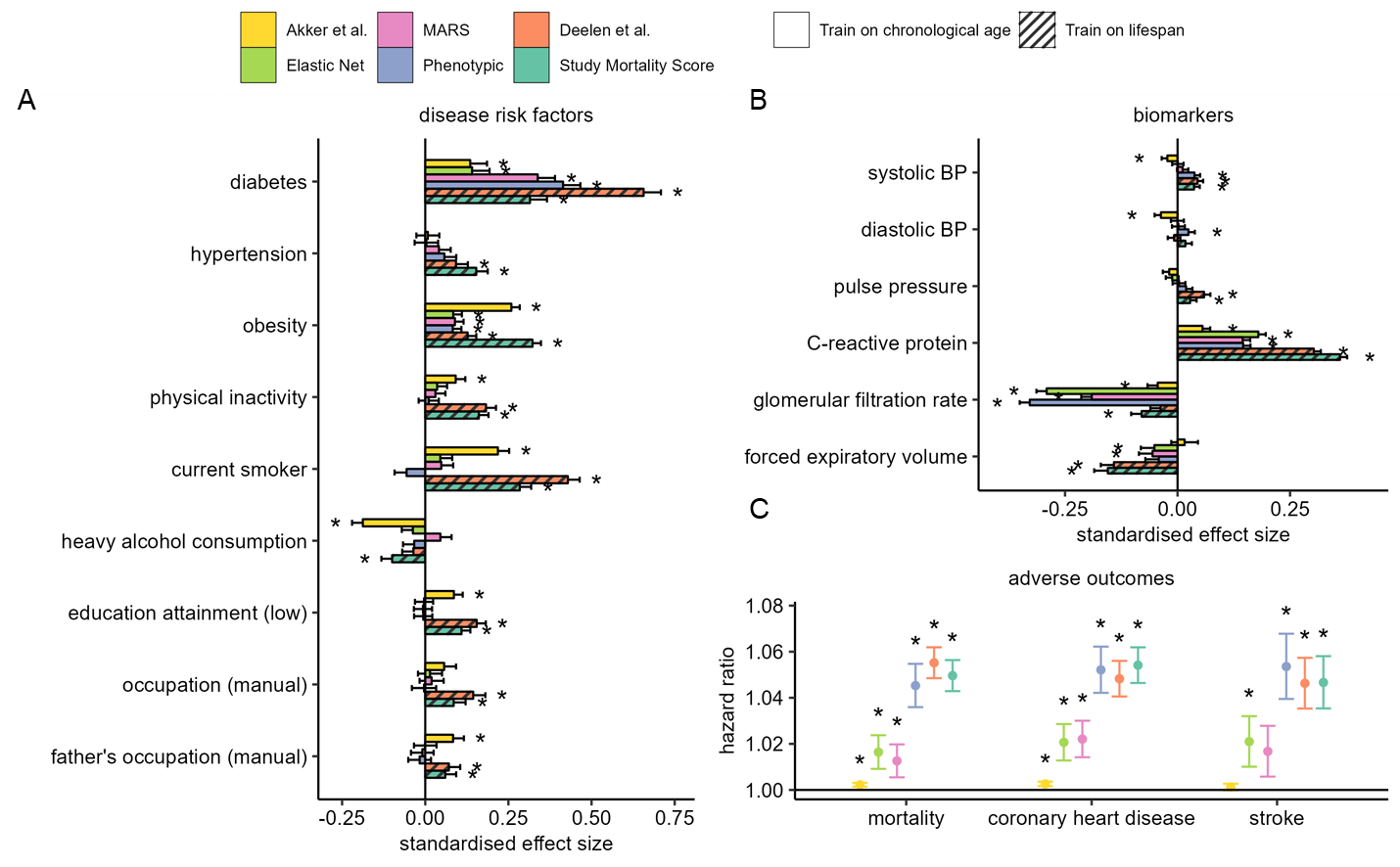


**Supplemental Figure 8** Model associations in UCLEB additionally adjusted for BMI *A) Associations with known disease risk factors. Estimates represent standard deviation (SD) change in metabolomic age associated with exposure which have been categorised into binary variables. B) Associations with age-related biomarkers. Estimates represent standard deviation change in metabolomic age associated with 1 SD unit change in biomarker levels. To avoid individuals from being accounted for more than once in the analysis, samples from YFS2001 and YFS2007, NFBC1966 (31y), and SABRE2 were excluded in the disease risk factor analysis, and subsequently up to 28,000 samples were included. C) Associations of metabolomic age models with adverse incident health events. Cox proportional regression models were adjusted for chronological age, sex, BMI and ethnicity.*


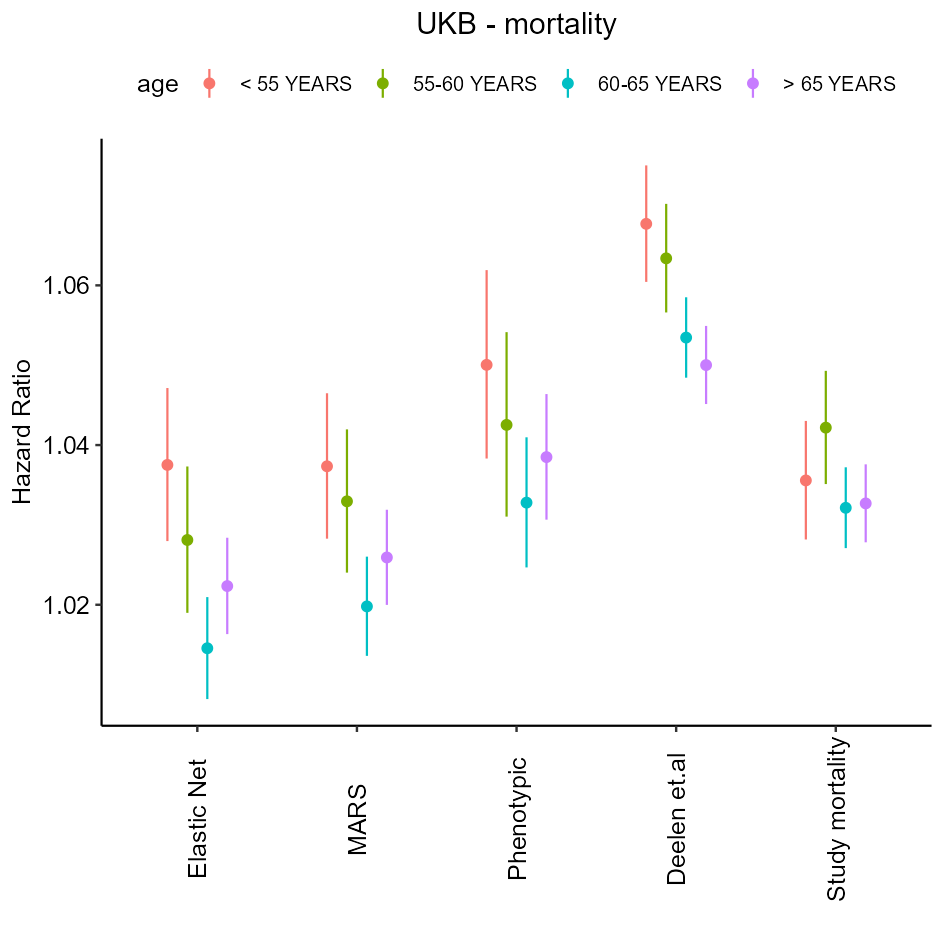


***Supplemental Figure 9*** Age stratified survival analyses for mortality in the UKB sample. Error bars represents the 95% confidence interval in the hazard ratios.
